# TRIM21 Dysfunction Enhances Aberrant B-Cell Differentiation in Autoimmune Pathogenesis

**DOI:** 10.3389/fimmu.2020.00098

**Published:** 2020-02-07

**Authors:** Yosuke Kunishita, Ryusuke Yoshimi, Reikou Kamiyama, Daiga Kishimoto, Koji Yoshida, Eijin Hashimoto, Takaaki Komiya, Natsuki Sakurai, Yumiko Sugiyama, Yohei Kirino, Keiko Ozato, Hideaki Nakajima

**Affiliations:** ^1^Department of Stem Cell and Immune Regulation, Yokohama City University Graduate School of Medicine, Yokohama, Japan; ^2^Center for Rheumatic Diseases, Yokohama City University Medical Center, Yokohama, Japan; ^3^Program in Genomics of Differentiation, National Institute of Child Health and Human Development, National Institute of Health, Bethesda, MD, United States

**Keywords:** TRIM21, systemic lupus erythematosus, B cell, immunoglobulin, lupus model mouse

## Abstract

TRIM21 is one of the autoantigens that reacts with an anti-SS-A antibody (Ab) present in patients with systemic lupus erythematosus (SLE) and Sjögren's syndrome. TRIM21 is thought to play a role in B-cell proliferation and apoptosis, among other activities. Here we examined a pathological role of TRIM21 in SLE. *Trim21*-deficient MRL/*lpr* mice were generated by backcrossing *Trim21*-deficient C57BL/6 mice to MRL/*lpr* mice. The levels of serum anti-dsDNA Ab and urine protein at 28 weeks of age were significantly higher in *Trim21*-deficient MRL/*lpr* mice as compared to wild-type MRL/*lpr* mice (*p* = 0.029 and 0.003, respectively). Resting B cells from *Trim21*-deficient mice showed significantly higher abilities to differentiate into plasmablasts and to produce Ab as compared with control mice. Due to the reduction of TRIM21-mediated ubiquitylation, IRF5 protein expression was increased in *Trim21*-deficient MRL/*lpr* mice (*p* = 0.021), which correlated with increased plasmablast generation and immunoglobulin production. B cells from SLE patients with anti-TRIM21 Ab seropositivity also showed a significantly higher ability to differentiate into plasmablasts as compared with those without anti-TRIM21 Ab or healthy controls. These results suggest that TRIM21 dysfunction contributes to SLE pathogenesis by promoting B-cell differentiation, for which anti-TRIM21 Ab may be partly responsible.

## Introduction

Systemic lupus erythematosus (SLE) is a chronic, systemic autoimmune disease of unknown cause. The etiology of SLE is thought to be multifactorial and to include contributions from both environmental factors, such as hormones (1), ultraviolet light (2), and infection (3), and genetic susceptibility which has been investigated by genome-wide association studies (4). As the disease is characterized by B-cell hyperactivity with the presence of increased immunoglobulin and a variety of autoantibodies in patients' sera, modulating B-cell function has been considered as a promising therapeutic approach (5, 6).

Tripartite motif containing 21 (TRIM21), a ~52-kDa protein also known as Ro52/SS-A, is one of major autoantigens recognized by anti-SS-A autoantibody present in sera of autoimmune diseases such as SLE and Sjögren's syndrome (7–9). TRIM21 belongs to the TRIM family, in which proteins are commonly comprised of a RING-finger domain, one or two B-box domains and a coiled-coil domain (10, 11).

Previous studies have revealed that TRIM21 is expressed ubiquitously but predominantly in hematopoietic cells and induced by type I and type II interferons (IFNs) (9, 11, 12). TRIM21 ubiquitylates IFN regulatory factors (IRFs) via its E3 ubiquitin ligase activity on the RING-finger domain and regulates the production of type I IFN and IL-12p40 (13–18). It has been reported that the TRIM21 overexpression in a B-cell line leads to reduced growth and increased apoptosis after activation (19, 20).

Two groups have disrupted the murine *Trim21* gene to analyze the function of TRIM21. We showed that NF-κB-dependent proinflammatory cytokine production is increased in *Trim21*-deficient C57BL/6 mice (17). Another group reported that IL-23-dependent Th17 differentiation and production of proinflammatory cytokines and type I IFN are enhanced and thereby contact dermatitis is evoked in *Trim21*-deficient C57BL/6 mice (16). These results suggest that TRIM21 has physiological roles for suppressing the excess inflammatory response and keeping homeostasis.

It has been shown that TRIM21 is expressed at higher levels in peripheral blood mononuclear cells (PBMCs) in SLE patients as compared to healthy controls (21). Although the role of TRIM21 in SLE pathogenesis is still unknown, previous studies strongly suggest that TRIM21 can act as a suppressor for autoimmune and inflammatory response and its dysfunction can cause the pathological state of SLE.

In this study, we generated and analyzed MRL/*lpr* lupus-prone mice in which *Trim21* gene is deleted to investigate the function of TRIM21 in autoimmune pathogenesis. The mice showed worsening SLE pathology with significantly increased autoantibody production and urine protein relative to wild-type MRL/*lpr* mice. We found the aberrant B-cell differentiation is accompanied by increased expression of transcription factors, IRF5 and BLIMP-1, which are crucial for B-cell differentiation and antibody (Ab) production (22–24). These factors have also been identified by SLE genome-wide association studies (25, 26). Similar to the results from mouse gene disruption studies, B cells from SLE patients with seropositivity of anti-TRIM21 Ab also indicated significantly higher ability to differentiate into plasmablasts and to produce Ab as compared with controls. Together, our results point that TRIM21 dysfunction promotes aberrant B-cell differentiation and Ab production in some SLE patients, which may be associated with anti-TRIM21 Ab.

## Materials and Methods

### Mice

*Trim21*^−/−^C57BL/6 mice have been described previously (17). C57BL/6JJmsSlc, MRL/MpJJmsSlc^+/+^ (MRL/+) and MRL/MpJJmsSlc^*lpr*/*lpr*^ (MRL/*lpr*) mice were purchased from Japan SLC (Hamamatsu, Japan). *Trim21*^−/−^C57BL/6 mice were backcrossed to MRL/*lpr* mice for more than 10 generations to produce *Trim21*^−/−^MRL/*lpr* mice. All mice were maintained under specific pathogen-free conditions within the animal facility at Yokohama City University, and female mice were used in all experiments. A comprehensive mouse genotyping examination was performed by ICLAS monitoring Center (Kawasaki, Japan). All experiments of skin-draining lymph nodes (sdLNs) were performed using bilateral axillary and inguinal lymph nodes. All animal experiment protocols were approved by the animal protocol ethics committee of Yokohama City University.

### Patients

Seventeen patients with SLE (16 women and one man), who fulfilled the revised 1997 American College of Rheumatology criteria for SLE (27), and five healthy controls (4 women and one man) were enrolled in the study. The study was conducted in accordance with the Declaration of Helsinki, and informed consent was obtained from all patients and healthy controls before study enrollment. The study design was approved by the ethics committee of Yokohama City University Hospital (B100701027).

### B Cell Preparation and Culture

Mice CD43^−^ resting B cells were isolated from the spleen of 8-week-old mice using Mouse B cell Isolation Kit (Miltenyi Biotec, Bergisch-Gladbach, Germany), according to the manufacturer's protocols. Isolated resting B cells were cultured in RPMI-1640 medium (Sigma-Aldrich, St. Louis, MO, USA) supplemented with 10% fetal bovine serum (MP Biomedicals, Santa Ana, CA, USA), 1 mM sodium pyruvate (Wako, Osaka, Japan), 10 mM HEPES (Gibco, Waltham, MA, USA), 100 μg/ml streptomycin, 100 U/ml penicillin (Gibco), and 1 mM 2-mercaptoethanol (Gibco). For some experiments, resting B cells were stimulated with 1 μg/ml anti-mouse IgM (Jackson ImmunoResearch, West Grove, PA, USA), 1 μg/ml anti-mouse CD40 (BioLegend, San Diego, CA, USA), 100 μg/ml poly(I:C) (Tocris Bioscience, Minneapolis, MN, USA), and/or 50 μg/ml imiquimod (AdooQ BioScience, Irvine, CA, USA). After incubation for 24 or 72 h, the cells were immediately used for flow cytometric analyses, and the supernatants were stocked at −80°C until use. The cell viability was assessed 24 h after stimulation using CellTiter-Blue Cell Viability Assay (Promega, Madison, WI, USA), according to the manufacturer's protocols.

Human PBMCs were separated by density gradient centrifugation using Lympholyte-H (Cedarlane, Burlington, Canada). Human CD43^−^ resting B cells were isolated from PBMCs using MojoSort Human B Cell (CD43^−^) Isolation Kit (BioLegend), according to the manufacturer's protocols. Isolated resting B cells were cultured in RPMI-1640 medium supplemented with 10% fetal bovine serum, 1 mM sodium pyruvate, 10 mM HEPES, 100 μg/ml streptomycin, 100 U/ml penicillin, and 1 mM 2-mercaptoethanol. For some experiments, resting B cells were stimulated with 1 μg/ml anti-human IgM Ab (BioLegend), 1 μg/ml recombinant human CD40L (BioLegend), 100 μg/ml poly(I:C), and/or 50 μg/ml imiquimod (R837; InvivoGen, San Diego, CA, USA). After incubation for 24 h, the cells were immediately used for flow cytometric analyses, and the supernatants were stocked at – 80°C until use.

### T Cell Preparation

Mice CD3^+^ T cells were isolated from the spleen and sdLN of 8-week-old mice using Mouse CD3ε MicroBead Kit (Miltenyi Biotec), according to the manufacturer's protocols. Gene expression of T-cell transcription factor in the isolated CD3^+^ T cells were evaluated using quantitative real-time RT-PCR (*q*PCR).

### *q*PCR

Total RNA was extracted with RNeasy Mini Kit (Qiagen, Hilden, Germany), and complementary DNA (cDNA) was prepared with SuperScript II Reverse Transcriptase (Invitrogen, Waltham, MA, USA). Fast SYBR Green Master Mix (Applied Biosystems, Waltham, MA, USA) was used for amplification of sample cDNA, and the data were analyzed by the StepOnePlus Real-Time PCR Systems (Applied Biosystems), according to the manufacturer's instructions. Transcript levels were standardized by the expression of *hypoxanthine phosphoribosyltransferase (Hprt)* mRNA. Primer sequences are listed in Table 1.

**Table 1:** Mouse primer list used in qPCR.

**Primer**	**Sequence**	**References**
*Trim21* forward	5′-CTGAGAAAGGGGAAAGAGTTGG-3′	In house
*Trim21* reverse	5′-GCCAGCAAGCTATTCTGAAGTG-3′	
*Tbet* forward	5′-CAACAACCCCTTTGCCAAAG-3′	(28)
*Tbet* reverse	5′-TCCCCCAAGCAGTTGACAGT-3′	
*Gata3* forward	5′-AGAACCGGCCCCTTATCAA-3′	(29)
*Gata3* reverse	5′-AGTTCGCGCAGGATGTCC-3′	
*Rorc* forward	5′-GGAGGACAGGGAGCCAAGTT-3′	(30)
*Rorc* reverse	5′-CCGTAGTGGATCCCAGATGACT-3′	
*Foxp3* forward	5′-GGCCCTTCTCCAGGACAGA-3′	(31)
*Foxp3* reverse	5′-GCTGATCATGGCTGGGTTGT-3′	
*Bcl6* forward	5′-CCTGCAACTGGAAGAAGTATAAG-3′	(32)
*Bcl6* reverse	5′-AGTATGGAGGCACATCTCTGTAT-3′	
*Blimp-1* forward	5′-GGCTCCACTACCCTTATCCTGGAGG-3′	(33)
*Blimp-1* reverse	5′-ACGCTGTACTCTCTCTTGGGGACAC-3′	
*Irf5* forward	5′-GGTCAACGGGGAAAAGAAACT-3′	Primer bank ID 6754368a1
*Irf5* reverse	5′-CATCCACCCCTTCAGTGTACT-3′	
*Irf4* forward	5′-GCCCAACAAGCTAGAAAG-3′	(34)
*Irf4* reverse	5′-TCTCTGAGGGTCTGGAAACT-3′	
*Irf8* forward	5′-GCATGAGCGAAGTTCCTGAGAT-3′	(35)
*Irf8* reverse	5′-CCATGTACTCATCCACAGAAGGTT-3′	
*Hprt* forward	5′-GATTAGCGATGATGAACCAGGTT-3′	In house
*Hprt* reverse	5′-CCTCCCATCTCCTTCATGACA-3′	

### Flow Cytometric Analysis

Spleen and sdLNs were collected from mice and passed through 40-μm nylon cell strainer (Falcon, Corning, NY, USA) to obtain a single cell. Red blood cells were removed by hemolysis using RBC Lysis Buffer (BioLegend), according to the manufacturer's protocols. The Fc of harvested cells and cultured B cells were blocked with anti-mouse CD16/CD32 Ab (BD Biosciences) for 10 min at 4°C. Then the cells were stained with Abs to surface markers for 20 min at 4°C. For intracellular staining, cultured B cells were treated with Fixation Buffer (BioLegend) and Intracellular Staining Permeabilization Wash Buffer (BioLegend), according to the manufacturer's protocols. After permeabilization, cells were stained with Abs to intracellular antigen for 20 min at 4°C.

For human PBMCs, CD43^−^ resting B cells, and cultured B cells, the FcR were blocked with anti-human FcR Ab (Human TruStain FcX; BioLegend) for 10 min at 4°C. The cell staining was performed according to the method of standardizing immunophenotyping for the human immunology project (36).

Zombie Aqua dye (BioLegend) or Zombie Green dye (BioLegend) were used for detecting dead cells in samples, according to the manufacturer's protocols. All gates were set using fluorescence minus one control. Stained cells were analyzed on BD FACSCanto II instrument (BD Biosciences, Franklin Lakes, NJ, USA), and data were analyzed using FlowJo software version 10 (FlowJo, Ashland, OR, USA).

### Multiple Soluble Analyte Immunoassay

The level of cytokines and immunoglobulins in cell supernatants were measured by bead-based immunoassay. The concentration of mice cytokines, immunoglobulins, and human immunoglobulins were measured using LEGENDplex Mouse Inflammation Panel, Mouse IFN-α Capture Bead, Mouse Anti-Virus Response Panel Standard, Mouse Anti-Virus Response Panel Detection Antibodies, Mouse Immunoglobulin Isotyping Panel and Human Immunoglobulin Isotyping Panel (BioLegend), according to the manufacturer's protocols.

### Measurement of Proteinuria and Serum Autoantibodies

Urine was collected from individual mice every 2 weeks from 8 weeks of age for over 16 h in metabolic cages (Shinano Manufacturing Co., Tokyo, Japan). The concentration of urine albumin and creatinine were measured using DC Protein Assay Reagent (Bio-Rad Laboratories, Hercules, CA, USA) and Creatinine Parameter Assay Kit (R&D Systems, Minneapolis, MN, USA), respectively, and the urine albumin-creatinine ratio was quantified.

Mouse serum anti-dsDNA Ab was measured using an enzyme-linked immunosorbent assay (ELISA) (Shibayagi, Shibukawa, Japan). Human serum anti-TRIM21 Ab was measured using an ELISA kit (Dr. Fooke Laboratorien, Neuss, Germany), according to the manufacturer's protocols.

### Western Blot Analysis

Mouse CD43^−^ resting B cells, which were stimulated with 1 μg/ml anti-mouse IgM Ab and 100 μg/ml poly(I:C) for 72 h, were lysed in RIPA buffer (50 mM Tris-HCl, 150 mM NaCl, 0.1% SDS, 0.5% sodium deoxycholate and 1% Nonidet P-40) in the presence of a protease inhibitor cocktail (Sigma-Aldrich). After 30 min incubation, the cells were sonicated by ultrasonication (Emerson Electric, St. Louis, MO, USA). Supernatants were collected after centrifugation at 15,000 g for 30 min and mixed with SDS sample buffer with 2-mercaptoethanol (Sigma-Aldrich). After 5 min boiling, the proteins were separated by NuPAGE 4–12% Bis-Tris gel electrophoresis (Invitrogen) and transferred onto polyvinylidene fluoride (PVDF) membrane (Merck Millipore). Anti-IRF4 (1:1,000 dilution, BioLegend), anti-IRF5 (1:1,000 dilution, Abcam, Cambridge, MA, USA), anti-IRF8 (1:1,000 dilution, Abcam), anti-ubiquitin (1:2,000 dilution, BioLegend), and anti-β-Actin antibodies (1:4,000 dilution, BioLegend) were used to detect the specific proteins. Chemiluminescence was developed using ECL Select Western Blotting Detection Reagent (GE Healthcare Life Sciences, Pittsburgh, PA, USA) and exposed to the LAS-3000 Mini Imaging System (FUJIFILM, Tokyo, Japan).

### Ubiquitylation Assay

Mice CD43^−^ resting B cells were stimulated with 1 μg/ml anti-mouse IgM Ab and 100 μg/ml poly(I:C) for 72 h. Cultures were treated with 25 μM MG-132 (Merck Millipore, Darmstadt, Germany) for 2 h before harvest. Extracts were immunoprecipitated with an anti-IRF5 Ab (Santa Cruz Biotechnology, Dallas, TX, USA) bound to protein G agarose beads (Cell Signaling Technology, Danvers, MA, USA). Immunoprecipitants were analyzed by Western blot using anti-ubiquitin Ab.

### Abs

Anti-CD3 (clone 17A2, catalog # 100227), anti-CD138 (clone 281-2, catalog # 142523), anti-CD317 (clone 927, catalog # 127023), anti-I-Ab (clone M5/114.15.2, catalog # 107631), anti-IgD (clone 11-26c.2a, catalog # 405725), anti-CD4 (clone RM4-5, catalog # 100539), anti-CD5 (clone 53-7.3, catalog # 100623), anti-CD8a (clone 53-6.7, catalog # 100733), anti-CD93 (clone AA4.1, catalog # 136511), anti-IgM (clone RMM-1, catalog # 406511), anti-B220 (clone RA3-6B2, catalog # 103235), anti-F4/80 (clone BM8, catalog # 123127), anti-CD1d (clone 1B1, catalog # 123521), anti-CD11b (clone M1/70, catalog # 101211), anti-CD11c (clone N418, catalog # 117309), anti-CD19 (clone 6D5, catalog # 115512), anti-Siglec H (clone 551, catalog # 129611), anti-Blimp-1 (clone 5E7, catalog # 150003) and anti-Bcl6 (clone 7D1, catalog # 358507) Abs were purchased from BioLegend and used for flow cytometric analyses of murine cells.

Anti-CD24 (clone ML5, catalog # 311105), anti-CD19 (clone HIB19, catalog # 302229), anti-CD27 (clone M-T271, catalog # 356411), anti-CD38 (clone HB-7, catalog # 356605), anti-CD20 (clone 2H7, catalog # 302357), anti-CD3 (clone UCHT1, catalog # 300433), and anti-IgD (clone IA6-2, catalog # 348220) Abs were purchased from BioLegend and used for flow cytometric analyses of human cells.

### Statistics

Statistical analysis was performed using Prism software version 6.0 (GraphPad Software, La Jolla, CA, USA) and SPSS Statistics software version 22 (IBM, Armonk, NY, USA). Unpaired two-tailed Student's *t*-test was used for parametric analysis of continuous variables. Chi-square test or Fisher's exact was used for analysis of categorical variables. A log-rank test was used for evaluating the survival curves statistically. A *p* < 0.05 was considered significant in all analyses.

## Results

### TRIM21 Deficiency Promotes Exacerbation of Lupus-Like Symptoms in MRL/*lpr* Mice

**Figure 1:**
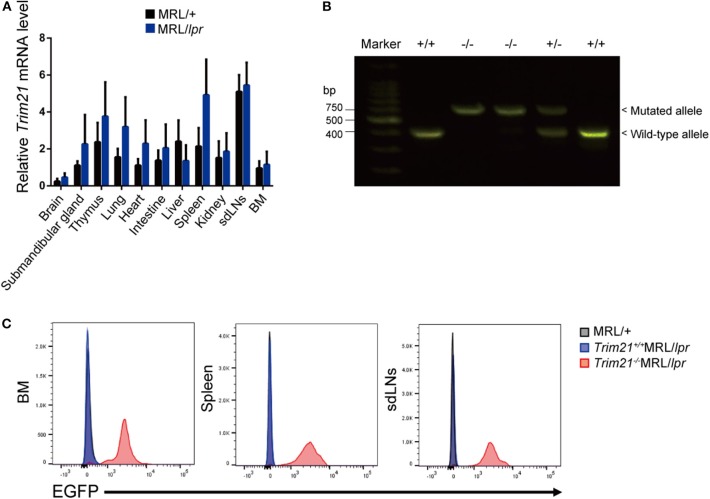
*Trim21* expression in tissues from MRL/*lpr* mice and generation of *Trim21*^−/−^MRL/*lpr* mice. **(A)** *Trim21* mRNA expression in tissues from MRL/+ and MRL/*lpr* mice at 8 weeks of age. (*n* = 4 in each group). **(B)** The genotype of *Trim21* is determined by PCR of genomic DNA from the tail. Arrows indicate the position of bands to detect the wild-type and mutated alleles. **(C)** Flow cytometric analyses reveal the EGFP expression by the mutated alleles in cells from BM, spleen and sdLNs of the 8-week-old *Trim21*^−/−^MRL/*lpr* mice. Data are representatives of at least three independent experiments. Values are shown as means ± SEM.

To verify the pathological role of TRIM21 in the autoimmune response, we first examined whether the expression of TRIM21 is influenced by an autoimmune condition. MRL/*lpr* mice, which are model mice spontaneously developing lupus-like symptoms, including anti-TRIM21 Ab production (37, 38), expressed the transcript of *Trim21* ubiquitously as shown in the previous study using C57BL/6 mice (17). However, the *Trim21* transcript tended to be expressed more preferably in lymphoid tissues such as thymus and spleen in MRL/*lpr* mice as compared to their control, MRL/+ mice (Figure 1A).

**Figure 2:**
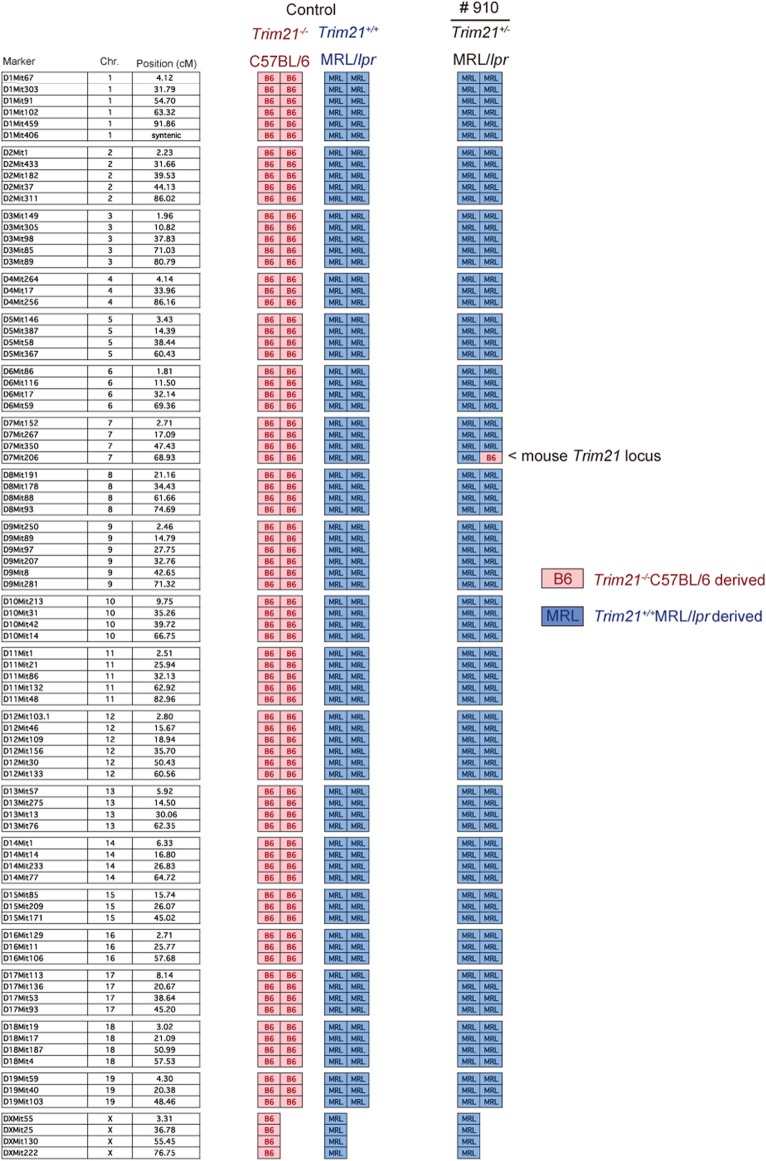
Genome scanning of backcrossed *Trim21*^+/−^MRL/*lpr* mice. A comprehensive examination of the genotype of *Trim21*^+/−^MRL/*lpr* mouse (Mouse # 910) generated by back-crossing for more than ten generations. Chr., chromosome.

The genetic background of *Trim21*^+/−^MRL/*lpr* mice was confirmed by genome scanning based on the microsatellite short tandem repeat analysis (Figure 2). The mice were genotyped by PCR analysis (Figure 1B), and *Trim21*-deleted allele was confirmed by the expression of an *enhanced GFP (EGFP)* reporter gene replacing *Trim21* exons in cells from the immune tissues using flow cytometric analysis (Figure 1C).

**Figure 3:**
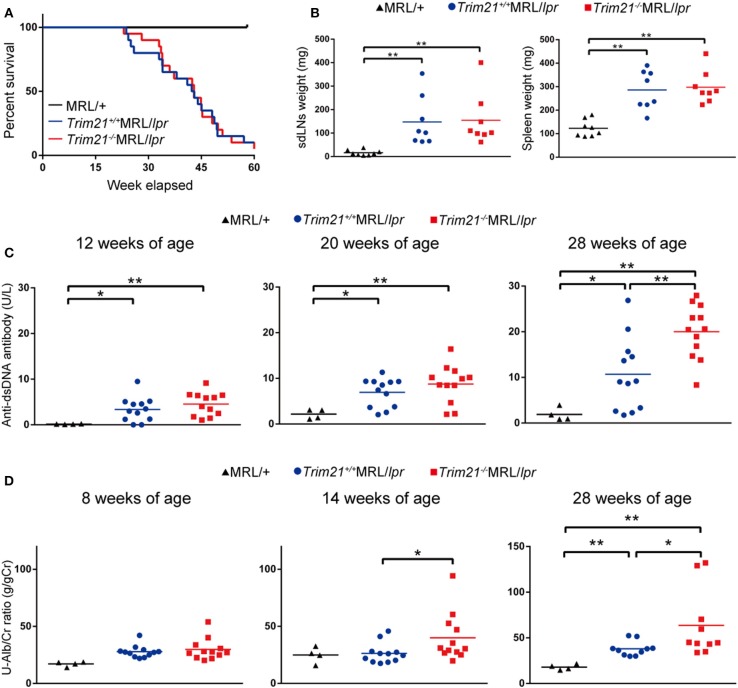
TRIM21 deficiency promotes exacerbation of lupus-like symptoms in MRL/*lpr* mice. **(A)** Survival rates of mice (MRL/+; *n* = 4, *Trim21*^+/+^MRL/*lpr*; *n* = 20 and *Trim21*^−/−^MRL/*lpr*; *n* = 20). **(B)** Weights of sdLNs and spleens at 10 weeks of age (*n* = 8 in each group). **(C)** Concentrations of serum anti-dsDNA Ab were measured by ELISA at 12, 20, and 28 weeks (MRL/+; *n* = 4, *Trim21*^+/+^MRL/*lpr*; *n* = 12 and *Trim21*^−/−^MRL/*lpr*; *n* = 12). **(D)** Proteinuria (albumin-creatinine ratio) was measured at 8, 14, and 28 weeks (MRL/+; *n* = 4, *Trim21*^+/+^MRL/*lpr*; *n* = 12 and *Trim21*^−/−^MRL/*lpr*; *n* = 12). Statistically significant data (*p < 0.05 and **p < 0.01) by Student's *t*-test, respectively.

As previously reported (39), *Fas*^*lpr*^ mutation shortened life-span (Figure 3A). Both *Trim21*^+/+^MRL/*lpr* and *Trim21*^−/−^MRL/*lpr* mice started to die at around 24 weeks of age and ~90% dead by 60 weeks of age. There was no significant difference in survival between them. Although splenomegaly and LN swelling were observed in both *Trim21*^+/+^MRL/*lpr* and *Trim21*^−/−^MRL/*lpr* mice, there was no significant difference in the weights of spleens and sdLNs between the two genotype mice (Figure 3B). On the other hand, the titer of serum anti-dsDNA Ab and daily urine protein were significantly increased over time in *Trim21*^−/−^MRL/*lpr* mice as compared with *Trim21*^+/+^MRL/*lpr* mice (Figures 3C,D). In particular, two severe cases of urine protein appeared in *Trim21*^−/−^MRL/*lpr* mice at 28 weeks while no such severe cases were seen in *Trim21*^+/+^MRL/*lpr* mice (Figure 3D).

Taken together, these results indicate that TRIM21 deficiency promotes exacerbation of systemic autoimmunity in MRL/*lpr* mice.

### TRIM21 Deficiency Increases B Cells in the Spleen of Lupus Model Mice

**Figure 4:**
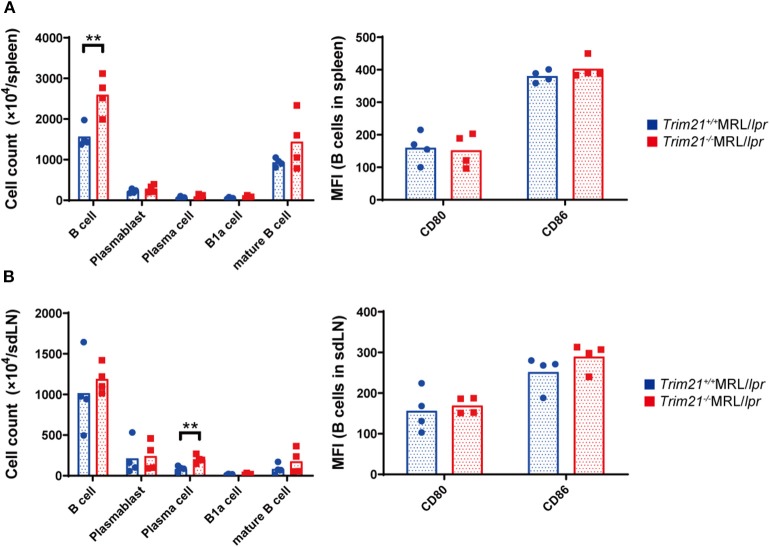
The comparison of B cell subset populations between *Trim21*^+/+^ and *Trim21*^−/−^MRL/*lpr* mice. Flow cytometric analyses and qPCR analyses were performed for spleen or sdLN cells at 10 weeks of age (*n* = 4 in each group). **(A,B)** B-cell subsets in spleens and sdLNs were classified by surface markers as follows: B cells; B220^+^CD19^+^, plasmablasts; CD19^+^CD138^+^, plasma cells; CD19^−^CD138^+^, B-1a cells; IgM^+^CD5^+^CD1d^−^ and mature B cells; IgM^+^IgD^+^CD19^+^. Mean fluorescence intensities (MFIs) of CD80 and CD86 were measured in B220^+^CD19^+^ B cells. Statistically significant data (**p < 0.01) by Student's *t*-test.

It has been reported that autoantibody production and urine protein due to glomerulonephritis in MRL/*lpr* mice depend on B cell-intrinsic MyD88 signal (40). To examine whether the autoimmune pathogenesis in *Trim21*^−/−^MRL/*lpr* mice is associated with B-cell abnormalities, we investigated the populations of leukocyte subsets in spleens and sdLNs by flow cytometric analysis. The number of B220^+^B19^+^ B cells was significantly higher in spleens from *Trim21*^−/−^MRL/*lpr* mice as compared to those from *Trim21*^+/+^MRL/*lpr* mice (*p* = 0.008; Figure 4A). The difference is mainly reflected by the increased number of IgM^+^IgD^+^CD19^+^ mature B cells in *Trim21*^−/−^MRL/*lpr* mice (Figure 4A). Although there was no significant difference in the number of B220^+^B19^+^ B cells in sdLNs between *Trim21*^+/+^MRL/*lpr* mice and *Trim21*^−/−^MRL/*lpr* mice, the number of CD19^−^CD138^+^ plasma cell was significantly higher in *Trim21*^−/−^MRL/*lpr* mice as compared to *Trim21*^+/+^MRL/*lpr* mice (*p* = 0.009; Figure 4B). There were no significant differences in the activation markers, CD80 and CD86, in B cells from spleens and sdLNs between *Trim21*^+/+^ and *Trim21*^−/−^MRL/*lpr* mice (Figures 4A,B).

**Figure 5:**
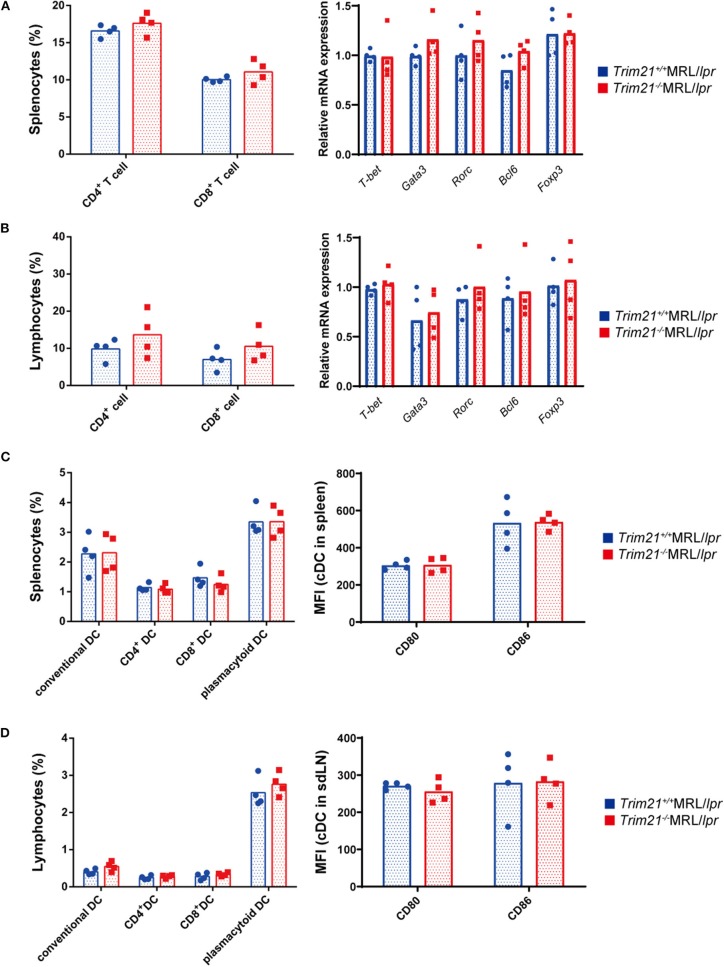
The comparison of T cell and DC subset populations between *Trim21*^+/+^ MRL/*lpr* mice and *Trim21*^−/−^MRL/*lpr* mice. Flow cytometric analyses and qPCR analyses were performed for sdLN cells at 10 weeks of age (*n* = 4 in each group). **(A,B)** T-cell subsets in spleen and sdLNs were classified by surface markers as follows: CD4^+^ T cell; CD3^+^CD4^+^ and CD8^+^ T cell; CD3^+^CD8^+^. mRNA expression levels of various T cell transcription factors in CD3^+^ T cells from spleens and sdLNs were measured by qPCR. **(C,D)** DC subsets in spleen and sdLNs were classified by surface markers as follows: cDC, I-Ab^+^B220^−^CD11c^+^; CD4^+^ DC, CD3^−^CD4^+^CD11c^+^; CD8^+^ DC, CD3^−^CD8^+^CD11c^+^; and pDC, CD317^+^B220^+^SiglecH^+^. MFIs of CD80 and CD86 were measured in cDCs.

It is known that various immune cells are activated due to an increase in abnormal autoreactive T cells in MRL/*lpr* mice (41). There was no significant difference in the proportion of CD4^+^ T cells and CD8^+^ T cells, and gene expressions of transcription factors related to Th1 (*Tbet*), Th2 (*Gata3*), Th17 (*Rorc*), Treg (*Foxp3*), and Tfh (*Bcl6*) subsets in CD3^+^ T cells from spleens and sdLNs between *Trim21*^+/+^MRL/*lpr* and *Trim21*^−/−^MRL/*lpr* mice (Figures 5A,B).

Dendritic cells (DCs) are also known to be important for autoimmune pathology like SLE. There were no significant differences in the proportions of conventional DCs (cDCs), CD4^+^ DCs, CD8^+^ DCs, and plasmacytoid DCs (pDCs), and the expression levels of activation markers, CD80 and CD86, in cDCs from spleens and sdLNs between *Trim21*^+/+^MRL/*lpr* and *Trim21*^−/−^MRL/*lpr* mice (Figures 5C,D).

These results suggested that B cells, rather than DCs and T cells, are essential in the autoimmune pathology of *Trim21*^−/−^MRL/*lpr* mice.

### TRIM21 Deficiency Promotes Aberrant B-Cell Differentiation and Ab Production in MRL/*lpr* Mice

**Figure 6:**
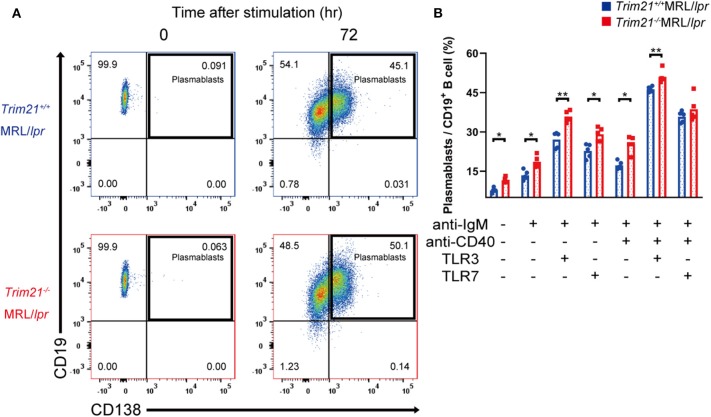
TRIM21 deficiency promotes aberrant B cell differentiation in MRL/*lpr* mice. **(A)** Flow cytometric analysis shows an increased rate of CD19^+^CD138^+^ plasmablasts at 72 h after stimulation with anti-IgM Ab and TLR3 ligand in *Trim21*^−/−^MRL/*lpr* mice. **(B)** Percentage of plasmablasts at 72 h after stimulation with anti-IgM Ab, anti-CD40 Ab, and/or TLR3/7 ligands (*n* = 4 in each group). Statistically significant data (*p < 0.05 and **p < 0.01) by Student's *t*-test. See also Supplemental Figure 1.

Although we found that mature B and plasma cells were increased in lymphoid tissues of *Trim21*^−/−^MRL/*lpr* mice, it is unknown whether they are due to an intrinsic abnormality of B cells or some abnormal state which can affect B-cell differentiation indirectly. To verify B cell-intrinsic function in *Trim21*^−/−^MRL/*lpr* mice, we isolated resting state B cells, which are CD43 negative, from spleens and stimulated them by BCR signal (anti-IgM Ab), CD40L signal (anti-CD40 Ab), and/or TLR3/7 ligands. Surprisingly, resting B cells from *Trim21*^−/−^MRL/*lpr* mice showed differentiation to plasmablasts in a significantly higher rate at 24 or 72 h after stimulation as compared to those from *Trim21*^+/+^MRL/*lpr* mice (Supplemental Figures 1A,B and Figures 6A,B). Not only in the absence but also in the presence of CD40L signal, known to be related to T cell-dependent B-cell activation, the B-cell differentiation into plasmablasts was enhanced by TRIM21 deficiency. The results suggest that TRIM21 has roles in B-cell differentiation both in T cell-dependent and T cell-independent manners [Figure 6B; (42, 43)].

There could be the possibility that the rate of plasmablasts was influenced by cell life-span because some previous reports showed that TRIM21 acts on apoptosis-related molecules such as BCL-2 and c-FLIP (19, 20). However, there was no significant difference in the survival of B cells at 24 h after stimulation between *Trim21*^+/+^MRL/*lpr* and *Trim21*^−/−^ MRL/*lpr* mice (Supplemental Figure 1C).

**Figure 7:**
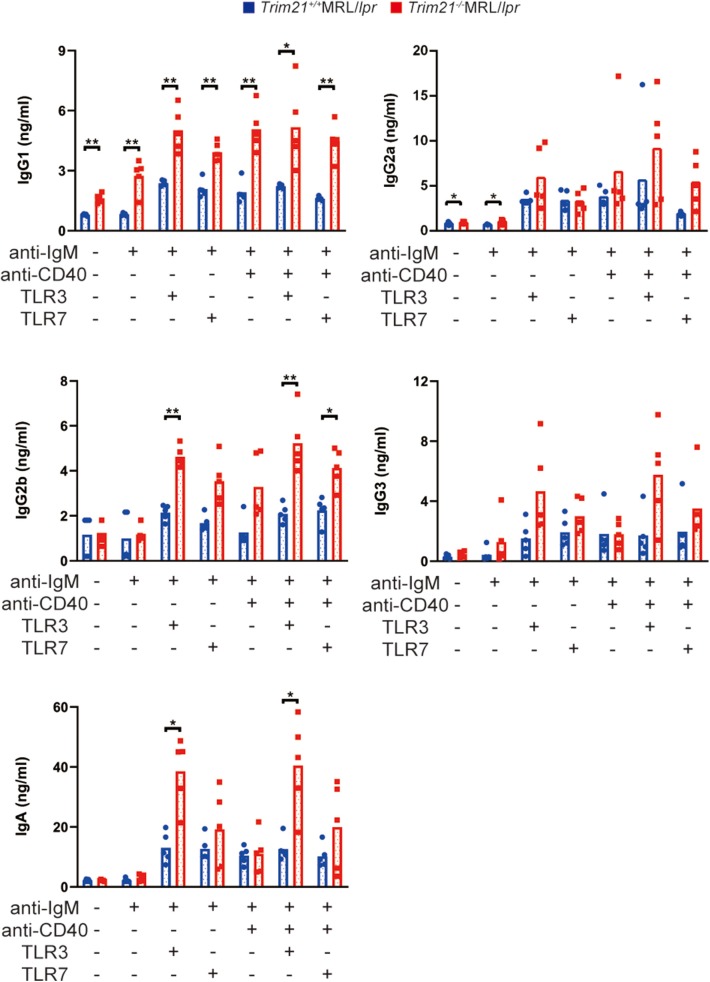
TRIM21 deficiency promotes aberrant Ab production in MRL/*lpr* mice. Concentrations of IgG1, IgG2a, IgG2b, IgG3, IgA, and IgM in culture supernatants of resting B cell stimulated with anti-IgM Ab, anti-CD40 Ab, and/or TLR3/7 ligands for 72 h were measured by multiple soluble analyte immunoassays (*n* = 4 in each group). Statistically significant data (*p < 0.05 and **p < 0.01) by Student's *t*-test, respectively. See also Supplemental Figure 2.

Next, we investigated the effect of TRIM21 deletion in Ab production by stimulating the resting B cells with anti-IgM Ab, anti-CD40 Ab, and/or TLR3/7 ligands. The concentrations of IgG1, IgG2a, and IgA in the cell supernatants, in which the stimulated resting B cells were cultured for 24 or 72 h, were significantly higher in *Trim21*^−/−^MRL/*lpr* mice as compared to *Trim21*^+/+^ MRL/*lpr* mice in multiple stimulation conditions (Supplemental Figure 2 and Figure 7).

Based on the above results, it is conceivable that TRIM21 deficiency leads to an intrinsic abnormality of B cells that induce aberrant B-cell differentiation and Ab production in MRL/*lpr* mice.

### TRIM21 Deficiency Induces Overexpression of *Blimp-1* and Suppression of *Bcl6* via Overexpression of IRF5 Due to the Decreased Ubiquitylation

IRF5 is the transcription factor known as one of the most essential factors for B-cell differentiation (23, 24). IRF5 promotes the differentiation of the late B cell and regulates transcription factors necessary for differentiation into activated B cells, plasmablasts and plasma cells (24). In the differentiation into antibody-producing cells (APCs) such as plasmablasts and plasma cells, IRF5 regulates the expression of IRF4, consequently promotes the expression of BLIMP-1, which drives mature B cells to differentiate into APCs, and thereby suppresses the expression of BCL-6, which is known as an inhibitor of APCs development (44). Furthermore, it has been reported that TRIM21 ubiquitylates IRF5 protein and promotes its degradation from the overexpression experiments using human embryonic kidney (HEK) 293 cell line (45).

**Figure 8:**
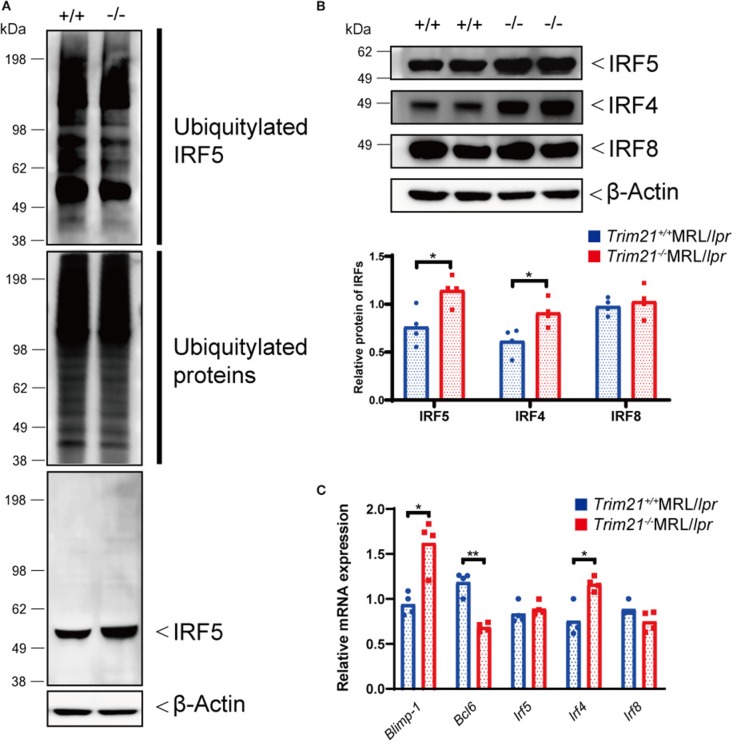
TRIM21 deficiency causes overexpression of IRF5 due to decreased ubiquitylation. **(A)** Extracts from resting B cells from *Trim21*^+/+^ and *Trim21*^−/−^MRL/*lpr* mice, which were stimulated with anti-IgM Ab and TLR3 ligand for 72 h, were immunoprecipitated with anti-IRF5 Ab and blotted with anti-ubiquitin Ab. Ubiquitylated IRF5 protein in the total cell lysate is decreased in the total extracts from *Trim21*^−/−^MRL/*lpr* mice as compared with *Trim21*^+/+^MRL/*lpr* mice (*top panel*). The amount of total ubiquitylated proteins is not different in two genotypes (*the second panel*). The amount of unubiquitylated IRF5 protein was higher in cells from *Trim21*^−/−^MRL/*lpr* mice as compared with *Trim21*^+/+^MRL/*lpr* mice (*the third panel*). β-Actin was used as a loading control (*the lowest panel*). **(B)** Western blot analysis shows that the expression level of IRF5, IRF4, and IRF8 protein in resting B cells at 72 h after stimulation with anti-IgM Ab and TLR3 ligand is higher in *Trim21*^−/−^MRL/*lpr* mice as compared to *Trim21*^+/+^MRL/*lpr* mice (*n* = 4 in each group). **(C)** mRNA expression levels of *Blimp-1, Bcl6, Irf5, Irf4*, and *Irf8* in resting B cells at 72 h after stimulation with anti-IgM Ab and TLR3 ligand (*n* = 4 in each group) in qPCR. Statistically significant (*p < 0.05 and **p < 0.01) data by Student's *t*-test, respectively.

Although the E3 ubiquitin ligase activity of TRIM21 for IRF5 is not known in primary B cells, we hypothesized that TRIM21 regulates plasma cell development via IRF5 ubiquitylation. In the ubiquitylation assay, there was no significant difference between *Trim21*^+/+^ and *Trim21*^−/−^ MRL/*lpr* mice in the total amount of ubiquitylated proteins in resting B cells at 72 h after stimulation with anti-IgM Ab and TLR3 ligand (Figure 8A). On the other hand, ubiquitylated IRF5 was remarkably decreased in *Trim21*^−/−^MRL/*lpr* mice as compared to *Trim21*^+/+^MRL/*lpr* mice (Figure 8A). Instead, the unubiquitylated IRF5 protein was significantly increased in *Trim21*^−/−^MRL/*lpr* mice as compared to *Trim21*^+/+^MRL/*lpr* mice (*p* = 0.021; Figures 8A,B). Furthermore, there was no significant difference in *Irf5* mRNA expression in resting B cells after stimulation with anti-IgM and TLR3 ligand for 72 h (Figure 8C), also suggesting that TRIM21 negatively regulates IRF5 protein expression via post-translational modifications.

**Figure 9:**
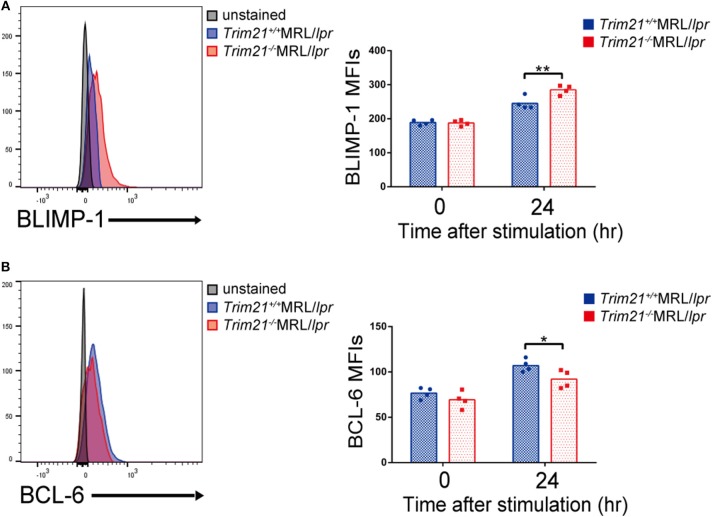
TRIM21 deficiency promotes BLIMP-1 overexpression and suppresses BCL-6 expression. **(A,B)** Flow cytometric analyses show that the intracellular expression level of BLIMP-1 is increased **(A)** and that of BCL-6 is decreased **(B)** by TRIM21 deficiency in resting B cells at 24 h after stimulation with anti-IgM Ab and TLR3 ligand (*n* = 4 in each group). Values are shown as means ± SEM. Statistically significance data (*p < 0.05 and **p < 0.01) by Student's *t*-test, respectively.

We further investigated the change in factors important for B-cell differentiation downstream region of IRF5 by *Trim21* deletion. IRF4 and IRF8 are also known to be essential transcription regulators in B-cell differentiation (44, 46, 47). *Irf4* has already been reported as one of the target genes of IRF5 (24). Both mRNA and protein of IRF4 showed significantly higher expression levels in B cells from *Trim21*^−/−^MRL/*lpr* mice at 72 h after stimulation as compared to those from *Trim21*^+/+^MRL/*lpr* mice (Figures 8B,C). On the other hand, there were no significant differences in the mRNA and protein expression levels of IRF8 between the two genotype mice (Figures 8B,C). IRF4 has already been shown to suppress BCL6 expression and promote BLIMP1 expression (48, 49). The mRNA expression of *Blimp1* was significantly increased, and the expression of *Bcl6* mRNA was significantly decreased in B cells from *Trim21*^−/−^MRL/*lpr* mice as compared to those from *Trim21*^+/+^MRL/*lpr* mice (Figure 8C). The flow cytometric analysis using intracellular staining showed significantly higher expression of BLIMP-1, and significantly lower expression of BCL-6 at the protein level in B cells from *Trim21*^−/−^MRL/*lpr* mice as compared to *Trim21*^+/+^MRL/*lpr* mice (*p* = 0.014 and 0.049, respectively; Figures 9A,B).

Together with the previous papers, these results suggest that TRIM21 negatively regulates the expression of IRF5 protein, decreases IRF4 expression, and thereby induces both BLIMP-1 suppression and BCL-6 expression, resulting in suppression of aberrant B-cell differentiation and Ab overproduction during lupus-like pathological condition.

### Aberrant B-Cell Differentiation and Ab Production in SLE Patients With Anti-TRIM21 Ab

Our previous study showed that *TRIM21* mRNA and protein in PBMCs of patients with SLE were significantly higher than those of healthy controls and that the expression level of *TRIM21* mRNA was well-correlated with disease activities of SLE (21). It is conceivable that the high expression of IFN signature in SLE may be responsible for increased expression of TRIM21 as its suppression mechanism. Furthermore, our previous study showed that a negative correlation is observed between the expression of IFN signature mRNA and *TRIM21* mRNA in healthy controls and SLE patients without anti-TRIM21 Ab who are considered to have normal function of TRIM21, but the correlation disappeared in SLE patients with anti-TRIM21 Ab (21). In this context, it was suggested that the function of TRIM21 is impaired by anti-TRIM21 Ab in patients with SLE. Therefore, we examined the difference of function of B cells between SLE patients with and without anti-TRIM21 Ab.

Seventeen patients with SLE and 5 healthy controls were enrolled in the study. Eleven and 6 of the patients showed seronegative and seropositive for anti-TRIM21 Ab, respectively. There were no significant differences in the background parameters including female ratio, age, disease duration, SLE activity, treatment, and laboratory data (Table 2).

**Table 2:** Demographic and clinical characteristics in human subjects.

	**Healthy controls *n = 5***	**SLE without anti-TRIM21 Ab *n = 11***	**SLE with anti-TRIM21 Ab *n = 6***	***p*-value**
**Demographic characteristics**				
Female, no. (%)	4 (80.0)	10 (90.9)	6 (100.0)	1.00
Age, year, mean ± SD	38.8 ± 10.2	39.7 ± 15.1	42.5 ± 17.5	0.75
**Clinical characteristics**				
Disease duration, year, mean ± SD		11.3 ± 7.8	11.5 ± 11.5	0.96
Total SLEDAI-2Kscore, mean ± SD		2.2 ± 2.0	1.7 ± 1.4	0.60
Sjögren's syndrome, no. (%)		1 (9.1)	2 (33.3)	0.51
**Medication**				
PSL, mg/day, mean ± SD		9.3 ± 5.4	7.4 ± 5.6	0.53
MMF, no. (%)		5 (45.5)	1 (16.7)	0.33
TAC, no. (%)		5 (45.5)	1 (16.7)	0.33
CyA, no. (%)		1 (9.1)	0 (0.0)	1.00
HCQ, no. (%)		6 (54.5)	4 (66.7)	1.00
MZB, no. (%)		0 (0.0)	1 (16.7)	0.35
**Laboratory data**				
WBC (/μl), mean ± SD		6,609 ± 1,983	6,217 ± 1,754	0.71
Lymphocyte (/μl), mean ± SD		810 ± 382	960 ± 312	0.45
Low complement, no. (%)		3 (27.3)	3 (50.0)	0.60
Increased DNA binding, no. (%)		4 (36.4)	2 (33.3)	1.00
Anti-nuclear Ab, no. (%)		9 (81.8)	5 (83.3)	1.00
Anti-TRIM21 Ab, no. (%)	0 (0.0)	0 (0.0)	6 (100.0)	< 0.01
Anti-SS-A Ab, no. (%)		6 (54.5)	5 (83.3)	0.33
Anti-SS-B Ab, no. (%)		1 (11.1)	1 (33.3)	1.00
Anti- Sm Ab, no. (%)		1 (11.1)	3 (50.0)	0.10
Anti-RNP Ab, no. (%)		3 (27.3)	3 (60.0)	0.60

*Analysis of p-value was compared between SLE without anti-TRIM21 Ab and SLE with anti-TRIM21 Ab by Student's t-test or chi-square test, respectively. PSL, prednisolone; MMF, mycophenolate mofetil; TAC, tacrolimus; CyA, cyclosporine A; HCQ, hydroxychloroquine; MZB, mizoribine; WBC, white blood cell count*.

**Figure 10:**
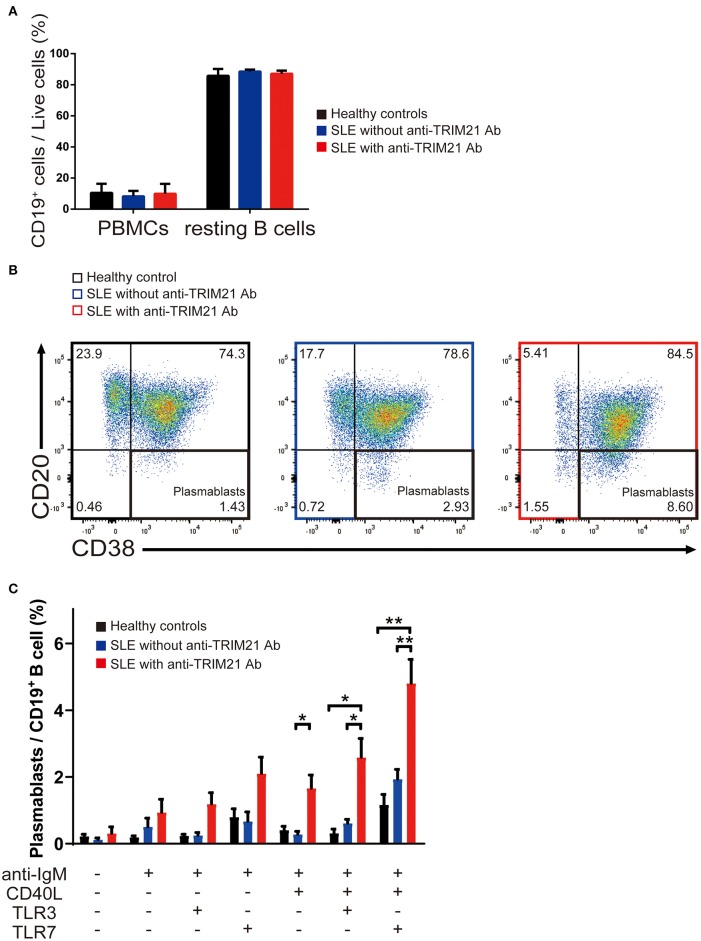
The seropositivity of anti-TRIM21 Ab is related to aberrant B cell differentiation in patients with SLE. **(A)** Percentages of CD19^+^ B cells in PBMCs and in resting B cells isolated by magnetic-activated cell sorting in SLE patients with anti-TRIM21 Ab, those without anti-TRIM21 Ab and healthy controls. **(B)** Flow cytometric analysis shows an increased rate of CD19^+^CD20^−^CD38^+^ plasmablasts at 24 h after stimulation with anti-IgM Ab, CD40L, and TLR7 ligand. The panels show the cells after gating with population positive to both CD19 and CD27. **(C)** Percentage of plasmablasts at 24 h after stimulation with anti-IgM Ab, CD40L, and/or TLR3/7 ligands in healthy controls (*n* = 5), SLE patients without anti-TRIM21 Ab seropositivity (*n* = 11) and those with anti-TRIM21 Ab seropositivity (*n* = 6). Values are shown as means ± SEM. Statistically significance data (*p < 0.05 and **p < 0.01) by Student's *t*-test, respectively.

There was no significant difference in the proportion of CD19^+^ B cells in PBMCs among healthy controls, SLE patients without anti-TRIM21 Ab and SLE patients with anti-TRIM21 Ab (Figure 10A). The proportion of CD19^+^ B cells after magnetically activated cell sorting of CD43^−^ resting B cell was more than 90% in all three groups (Figure 10A).

**Figure 11:**
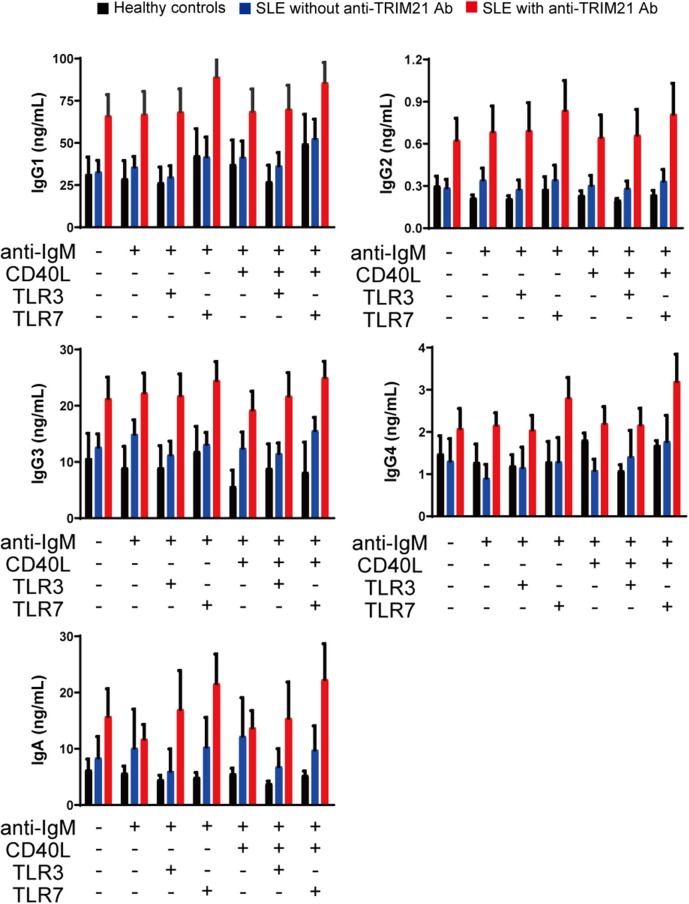
The immunoglobulin productivity in patients with SLE. Concentrations of IgG1, IgG2, IgG3, IgG4, IgA, and IgM in culture supernatants of human resting B cells stimulated with anti-IgM Ab, CD40L and/or TLR3/7 ligands were measured by multiple soluble analyte immunoassays (healthy controls; *n* = 5, SLE patients without anti-TRIM21 Ab; *n* = 11 and SLE patients with anti-TRIM21 Ab; *n* = 6). Values are shown as means ± SEM.

CD43^−^ resting B cell was stimulated by BCR signal (anti-IgM Ab), CD40L, and/or TLR3/7 ligands for 24 h and then tested for differentiation into CD19^+^CD20^−^CD38^+^ plasmablasts. As shown in Figures 10B,C, the percentage of plasmablasts was the highest in SLE patients with anti-TRIM21 Ab. Further, the percentage of plasmablasts was significantly higher in SLE patients without anti-TRIM21 Ab than that in healthy controls. Accordingly, the amounts of immunoglobulins produced in the culture was the highest in SLE patients with anti-TRIM21 Ab, followed by B-cell cultures from SLE patients without the Ab, and the lowest in healthy controls (Figure 11).

These results indicate that B cells in patients with SLE, especially in those with anti-TRIM21 Ab, are defective in plasmablasts differentiation and Ab production. Anti-TRIM21 Ab may be related to the B-cell abnormalities via inhibition of TRIM21 function.

## Discussion

TRIM21 has been shown to regulate the expression of various proinflammatory cytokine genes, type I IFN genes and IFN-inducible genes by modulating the signals from the innate immunoreceptor such as TLR via ubiquitylation of various IRF proteins (12, 45, 50). Previous gene disruption studies have not shown the pathological role of TRIM21 in an autoimmune state such as SLE (16, 17). Therefore, we made *Trim21*^−/−^MRL/*lpr* mouse widely used as a lupus mouse model and examined the phenotype change and the reactivity of immune cells to various TLR ligands (37). This is the first report that shows the pathological role of TRIM21 dysfunction in the lupus mouse model.

Flow cytometric analyses showed that B cells were significantly increased in the spleens and sdLNs by *Trim21* gene disruption in lupus-prone mice. Interestingly, Brauner et al. reported that the prognosis was reduced when the *Trim21* gene expression was decreased in patients with diffuse large B-cell lymphoma (51). The report suggests that the dysfunction of TRIM21 promotes abnormal activation and proliferation of B cells. Lazzari et al., showed by the overexpression experiments using cell lines that TRIM21 regulates the expression of IRF5 by ubiquitylation (45). IRF5 has been shown to be necessary for B-cell differentiation and Ab production of autoreactive B cells (23, 24, 52). The knockdown of IRF5 by siRNA transfection in human primary naïve B cells reduces plasmablast differentiation and Ab production (24). Our data are well-consistent with these previous studies because our ubiquitylation assay showed that TRIM21 deficiency leads to decreased ubiquitylation and increased expression of IRF5 in B cells. On the other hand, a recent study showed that the *Irf5* risk alleles for SLE intrinsically affects the function of myeloid cells rather than that of B cells (53). Although the overexpression experiments are considered as somewhat artificial in general, not the risk haplotype but the levels of IRF5 expression may be important in B cell function.

We found the differences in B-cell differentiation and Ab production between the *Trim21*^−/−^MRL/*lpr* mice and the *Trim21*^+/+^MRL/*lpr* mice. B-cell activation can occur both in T cell-dependent and T cell-independent manners and DCs also play a role in B-cell differentiation via several functions including the production of B cell-activating factor belonging to the tumor necrosis factor family (BAFF) (54, 55). In our experiments, *Trim21* deletion did not change the states of T cells and DCs. Moreover, we did not find any differences in the expression of CD80 and CD86, which are important for T cell-B cell interaction (56), in B cells between *Trim21*^+/+^ and *Trim21*^−/−^MRL/*lpr* mice. These results suggest that the enhanced B-cell proliferation and Ab production by *Trim21* deletion is mainly caused by the abnormality of B cells themselves, and not by that of T cells or DCs.

Anti-SS-A Ab is observed in sera of patients with autoimmune diseases such as SLE and Sjögren's syndrome, and TRIM21 is one of the autoantigens that bind to anti-SS-A Ab (57, 58). We have shown that negative correlation between *TRIM21* and type I IFNs expression, which is seen in PBMCs from healthy controls, disappeared in those from SLE patients, especially those with anti-TRIM21 Ab seropositivity (21). In this study, the differentiation from resting B cells to plasmablasts and Ab production were significantly increased in SLE patients with anti-TRIM21 Ab seropositivity as compared to those negative for anti-TRIM21 Ab. In combination with previously reported *in vitro* data that TRIM21 has a suppressive role in human B-cell proliferation (19, 20, 59), these results strongly suggest that the disruption of TRIM21 fu0nction can be caused partly by anti-TRIM21 Ab in patients with SLE. Consistent with this hypothesis, B-cell differentiation and Ab production were increased in the *Trim21*^−/−^ MRL/*lpr* mice as compared with the *Trim21*^+/+^ MRL/*lpr* mice in our experiments. On the other hand, type I IFNs induce TRIM21 expression while TRIM21 itself suppresses the production of type I IFNs (14, 15, 17, 60). This suggests that the increased TRIM21 expression in SLE patients seems due to an increase in serum type I IFN concentration, and it can be considered as a negative feedback reaction to suppress type I IFN overproduction. Therefore, further increasing TRIM21 expression and suppressing the production of anti-TRIM21 Ab can be new treatment strategies for SLE.

How the extracellular anti-TRIM21 Ab can affect the function of intracellular TRIM21 proteins is an important point that has not been studied sufficiently yet. Further studies are needed to clarify this issue. It is possible that the extracellular anti-TRIM21 Ab directly invades the cell and binds to intracellular TRIM21, which may affect function. There were some reports that an anti-DNA Ab that recognizes DNA in the nucleus invades intracellularly from outside the cell via calreticulin or myosin-1 (61–63). Furthermore, it was recently reported that TRIM21 recruits incoming antibody-coated virus and targets it to the proteasome via its E3 ligase activity, resulting in degradation of virions in the cytosol of the infected cells (64). Another recent report shows that anti-TRIM21 autoantibodies inhibit the E3 ligase activity of TRIM21 by sterically blocking the E2/E3 interaction between TRIM21 and UBE2E1 (57). Thus, there is a possibility that serum anti-TRIM21 Ab enters the cytosolic compartment by some cue, interacts with TRIM21 protein, and interferes with its function.

In our experiment, *Trim21*^−/−^MRL/*lpr* mice showed significant increases in urinary protein and serum dsDNA Ab titers as compared to wild-type MRL/*lpr* mice. Therefore, it is certain that the *Trim21* gene deletion has an impact on nephritis and autoantibody production in lupus pathogenesis. On the other hand, *Trim21*^−/−^MRL/*lpr* mice were not different from wild-type mice in the survival curve. The result suggests that various compensatory actions work against the changes associated with the *Trim21* deletions to maintain life as a whole body. This may also be related to the fact that human SLE is a multifactorial disease.

During the preparation of this paper, Brauner et al. have reported that follicular B cells and Ab production were significantly increased by immunization of *Trim21*-deficient C57BL/6 mice with ovalbumin (65). Although the paper does not show an effect of TRIM21 deficiency on phenotype in disease model and the mechanism of B cell increase has not been investigated, their data are well-consistent with our results.

In conclusion, we showed that TRIM21 deficiency promotes aberrant B-cell differentiation and Ab production, leading to exacerbation of the lupus-like symptoms in lupus-prone mice. We also found that B cells from SLE patients with anti-TRIM21 Ab also have significantly higher abilities to differentiate into plasmablasts and to product Ab, suggesting that anti-TRIM21 Ab may be related to the TRIM21 dysfunction in human SLE pathogenesis. These findings suggest that TRIM21 and anti-TRIM21 Ab can be promising targets for SLE treatment.

## Data Availability Statement

The datasets generated for this study are available on request to the corresponding author.

## Ethics Statement

The studies involving human participants were reviewed and approved by the ethics committee of Yokohama City University Hospital (B100701027). The patients/participants provided their written informed consent to participate in this study. This animal study was reviewed and approved by the Animal Protocol Ethics Committee of Yokohama City University.

## Author Contributions

YKu performed experiments with assistance from RY, RK, DK, KY, EH, TK, and NS. YKu, RK, and DK backcrossed and generated the *Trim21*^−/−^MRL/*lpr* mice. YKu and RY conceived the project, designed the experiments, analyzed the results, prepared figures, and devised the manuscript. YS, YKi, KO, and HN coordinated and critically discussed research work. All the above-listed authors edited the manuscript.

### Conflict of Interest

The authors declare that the research was conducted in the absence of any commercial or financial relationships that could be construed as a potential conflict of interest.
