# Supplementary material for: TRIM21 Dysfunction Enhances Aberrant B-Cell Differentiation in Autoimmune Pathogenesis
Source: Front Immunol. 2020 Feb 7;11:98. doi: 10.3389/fimmu.2020.00098 (PMC7020776; doi:10.3389/fimmu.2020.00098)
Supplement: Supplementary file 1 [file Image_1.pdf]

Supplemental Figure 1. TRIM21 deficiency promotes aberrant B-cell differentiation in MRL/*lpr* mice.

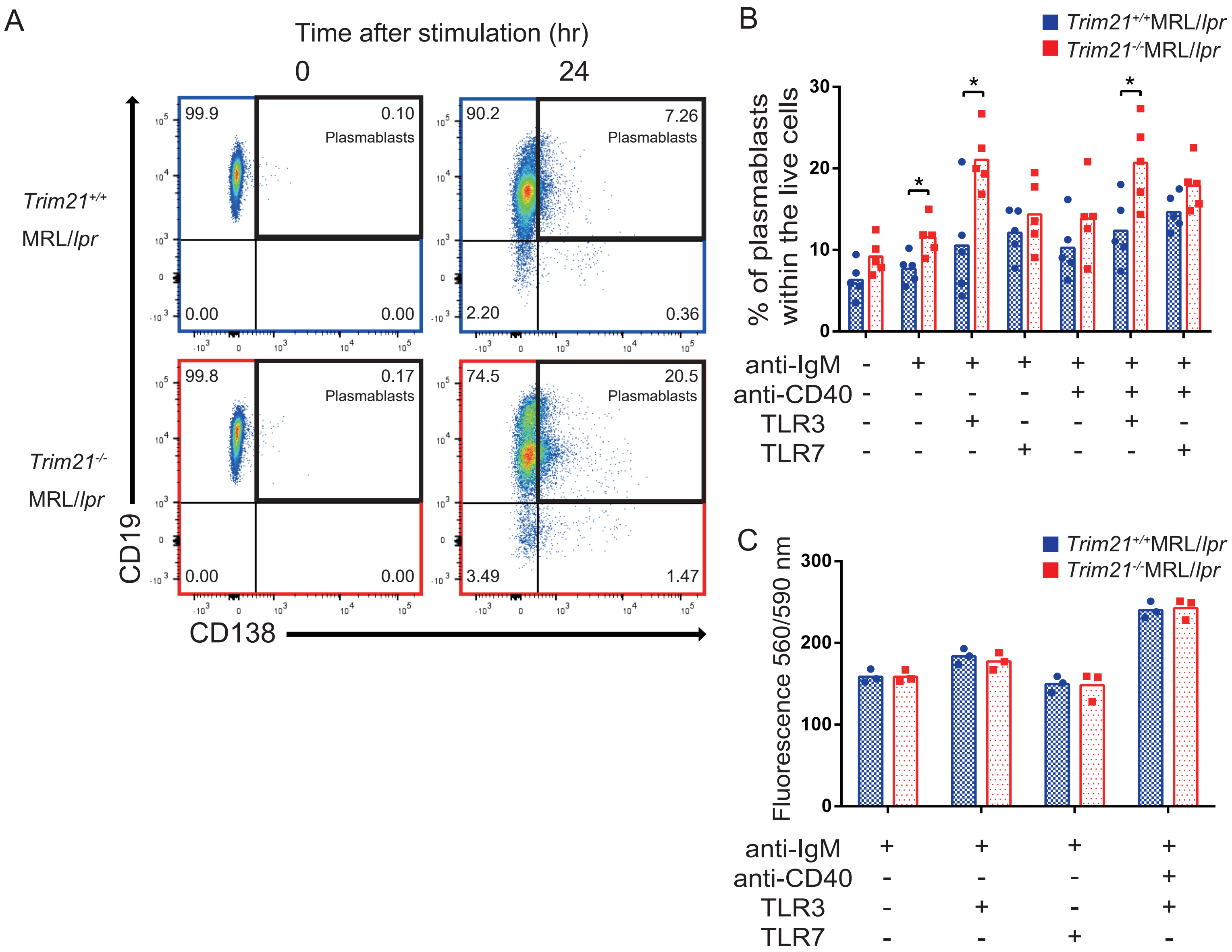

(A) Flow cytometric analysis shows an increased rate of $CD19^+CD138^+$ plasmablasts at 24 hours after stimulation with anti-IgM Ab and TLR3 ligand in *Trim21*$^{-/-}$MRL/*lpr* mice. (B) Percentage of plasmablasts at 24 hours after stimulation with anti-IgM Ab, anti-CD40 Ab and/or TLR3/7 ligands (n = 4 in each group). (C) Cell viability assay shows no significant difference between *Trim21*$^{+/+}$ and *Trim21*$^{-/-}$MRL/*lpr* mice at 24 hours after stimulation (n = 3 in each group). Statistically significant data (*, $p < 0.05$) by Student' s *t*-test.
