# Supplementary material for: TRIM21 Dysfunction Enhances Aberrant B-Cell Differentiation in Autoimmune Pathogenesis
Source: Front Immunol. 2020 Feb 7;11:98. doi: 10.3389/fimmu.2020.00098 (PMC7020776; doi:10.3389/fimmu.2020.00098)
Supplement: Supplementary file 2 [file Image_2.pdf]

Supplemental Figure 2. TRIM21 deficiency promotes aberrant Ab production in MRL/*lpr* mice.

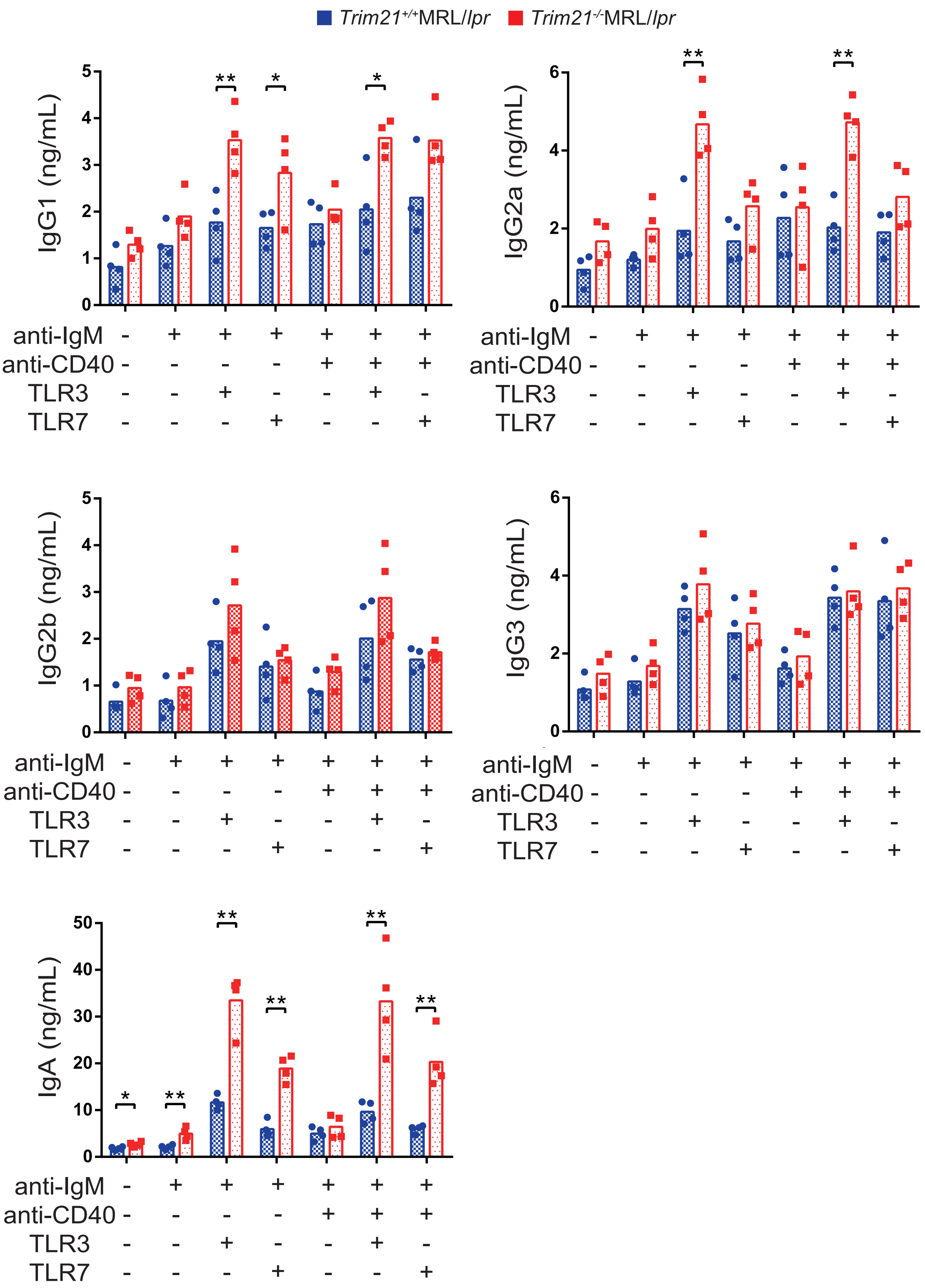

Concentrations of IgG1, IgG2a, IgG2b, IgG3, IgA and IgM in culture supernatants of resting B cell stimulated with anti-IgM Ab, anti-CD40 Ab and/or TLR3/7 ligands were measured by multiple soluble analyte immunoassays (n = 4 in each group). Statistically significant data (*, $p < 0.05$ and **, $p < 0.01$) by Student' s *t*-test, respectively.
